# Thermal Characteristics of Selected Phosphate Ores and the Effect of Inorganic Salts on Their Calcination

**DOI:** 10.1021/acsomega.3c05573

**Published:** 2023-11-17

**Authors:** Jakub Zieliński, Marcin Biegun, Maciej Kaniewski, Marta Huculak-Mączka, Józef Hoffmann

**Affiliations:** Department of Engineering and Technology of Chemical Processes, Faculty of Chemistry, Wrocław University of Science and Technology, ul. M. Smoluchowskiego 25, 50-372 Wrocław, Poland

## Abstract

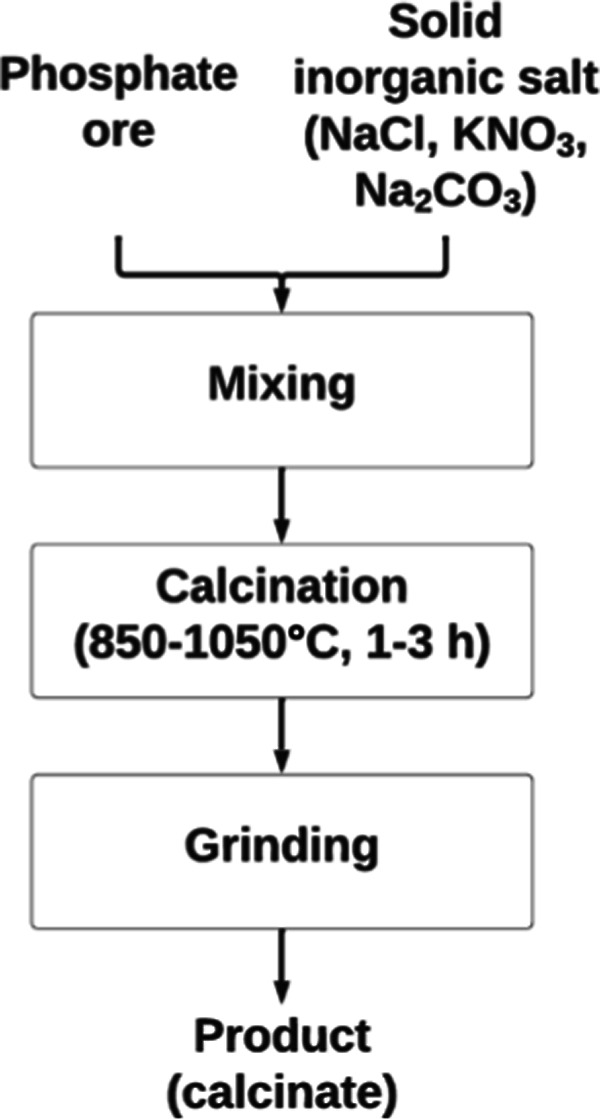

Calcination of phosphate ore is one of the methods of ore processing, i.e., increasing the phosphorus content (P_2_O_5_) in the ore. However, this process is very energy-intensive and not economically justified in most cases. It can be improved by using additives to lower the required calcination temperature. In this work, several samples of phosphate ores were subjected to thermal analysis using thermogravimetry coupled with mass spectrometry (TG-MS) to study their behavior during the calcination process. Then, selected phosphate ore from the Tunisian deposit was mixed with NaCl, KNO_3_, or Na_2_CO_3_ and calcined in various regimes (temperature and time). Uncalcined samples, together with obtained calcinates, were also subjected to thermal analysis by TG-MS. Temperature ranges in which the mass loss occurred were defined and discussed. Appropriate models of sample weight loss were derived and visualized by using the response surface methodology. Explanations of possible processes observed during the heating of phosphate ore samples with inorganic salt addition were proposed.

## 1 Introduction

Phosphate ores are the only industrially used raw materials for the production of phosphate fertilizers. Due to the chemistry of technologies used within the fertilizer industry (direct production from ore or indirect via phosphoric acid as an intermediate product), most of the impurities present in the original ore are transferred to the final product. Those impurities are, among others: arsenic, cadmium, chromium, copper, uranium, and zinc.

Cadmium compounds are always present in conjunction with phosphorus in minerals. Its content typically ranges from 1 to 100 mg/kg (0.0001–0.01 wt %) in the ore. Therefore, the amount of cadmium processed and entering the environment is significant. Cadmium is highly toxic to humans. Long-term exposure to cadmium through air, food, soil, and water leads to cancer and organ system toxicity such as cardiovascular, central/peripheral nervous, reproductive, respiratory, skeletal, and urinary systems.^1^

Calcination^2,3^ and solvent extraction from phosphate ore^4,5^ or phosphoric acid^6−11^ are three basic methods for the decadmiation of phosphate fertilizers that are commonly listed in the literature. Neither of them is currently used in the industry due to their high cost. However, calcination of phosphate ore is a well-known method for ore processing.^12−17^ Although it is very energy-consuming and therefore not economically justified in most cases, some reports have shown that it can significantly lower the cadmium content in the ore. The process can be further improved with additives (e.g., Cl^–^ ions) that lower the required calcination temperature.^18^

Several calcination processes to beneficiate phosphate ore have been tested on an industrial scale in the past. Probably, the best known is the CERPHOS process, developed in Morocco in the 1980s. At that time, a calcination plant for the removal of cadmium from phosphate ore operated by Nauru Phosphate Corporation was also established. However, the plant has already been closed, and no industrial plants using calcination to purify raw materials are currently known.^19^

The aim of the study was to characterize the calcination process by exploring phenomena occurring during the heating of phosphate ores and their systems with various inorganic additives. For this purpose, several phosphate ores were subjected to TG-MS thermal analysis. The phosphate ore obtained from the Tunisian deposit was then selected and subjected to calcination with different additives (NaCl, KNO_3_, Na_2_CO_3_) in various calcination regimes (temperature 850–1050 °C, time 1–3 h). Calcinates as well as uncalcined raw materials were subjected to TG-MS thermal analysis.

## 2 Materials and Methods

All phosphate ore samples used in this study were air-dried under ambient conditions, then ground in an IKA A11 Basic analytical mill, and sieved to a fraction smaller than 300 μm prior to any experiments.

The chemical composition of samples was analyzed by flame atomic absorption spectrometry (FAAS). The Thermo Scientific iCE 3000 Series instrument with a 50 mm universal burner was used in the measurements.

Phosphate ore samples were subjected to thermogravimetry coupled to mass spectrometry (TG-MS). The NETZSCH STA 449 F3 Jupiter thermal analyzer, with thermobalance, and the NETZSCH Aeolos QMS 403C quadrupole mass spectrometer were used. The sample containing 20 mg of the studied material was placed in an 85 μL crucible made of alumina and then heated to a temperature of 1100 °C with a heating rate of 10 °C/min in synthetic air with a total flow of 30 mL/min. Measurements were preceded by correction measurements up to 1200 °C to compensate for thermal effects associated with the characteristics of the crucible.

Based on the TG-MS measurements, phosphate ore from the Tunisian deposit was selected for further calcination studies. Samples of the ore were mixed with solid NaCl, KNO_3_, and Na_2_CO_3_ in a 19:1 (m/m) ratio. The mixtures, as well as the original ore, were then calcined in porcelain crucibles in the FCF 12 SHM resistance muffle furnace at temperatures (850, 950, 1050 °C) and calcination time (1, 2, and 3 h) according to the experiment matrix of the full factorial design plan. Approximately 10 g of the sample was placed in the crucible, which was inserted into a preheated oven, kept for the selected time, and then removed and cooled in a desiccator. The calcined products were then ground in an IKA A11 Basic analytical mill and sieved to a fraction smaller than 300 μm.

Results obtained during experiments were analyzed by using Statistica computational software. Graphs presented in this paper were prepared by using Origin software.

## 3 Results and Discussion

### 3.1 Thermal Analysis of Phosphate Ores

**Figure 1:**
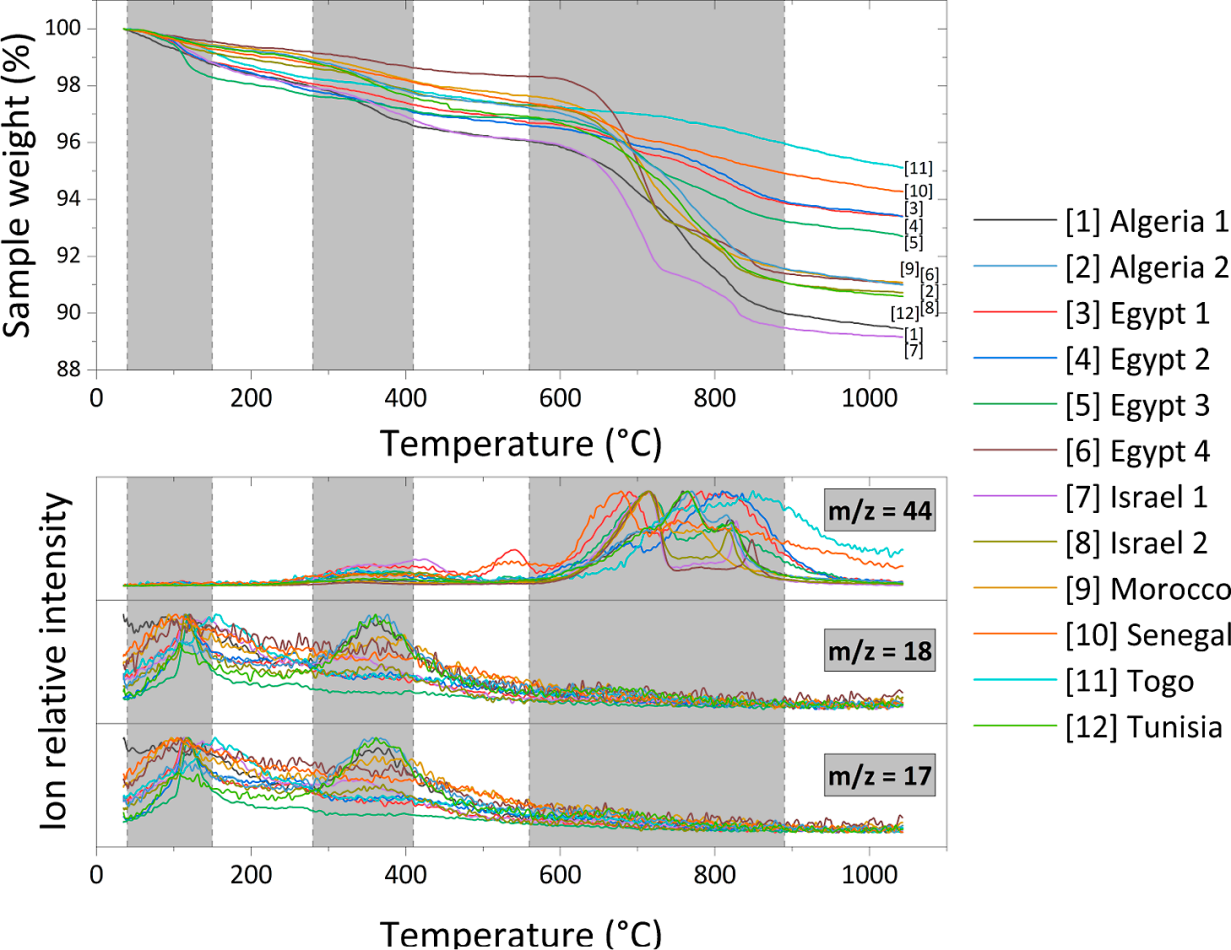
TG and MS curves for the phosphate ore samples.

Samples of phosphate ores from various deposits were subjected to TG-MS. The chemical composition of ores is provided in Table 1, while selected data points from thermogravimetry curves for each sample are presented in Table 2 and all results, together with MS curves for *m*/*z* 17, 18, and 44, are presented in Figure 1.

**Table 1:** Chemical Composition of the Phosphate Ore Samples

phosphate ore	component content [wt %]				
	P_2_O_5_	CaO	MgO	Al_2_O_3_	Fe_2_O_3_
Algeria 1	30.8	47.7	0.67	1.14	1.22
Algeria 2	37.9	54.8	1.08	0.59	0.45
Egypt 1	30.1	44.7	0.50	1.05	1.01
Egypt 2	29.3	44.9	0.42	1.14	1.15
Egypt 3	27.4	42.8	0.33	1.23	1.30
Egypt 4	35.4	53.1	0.21	0.28	0.41
Israel 1	30.8	48.3	0.35	0.09	0.37
Israel 2	35.7	52.0	0.46	0.24	0.42
Morocco	27.7	42.8	0.42	0.32	0.28
Senegal	34.4	50.7	0.56	1.06	1.08
Togo	34.6	50.5	1.67	0.38	0.24
Tunisia	26.2	46.4	0.85	0.38	0.49

**Table 2:** Selected Data Points from Thermogravimetry Curves of the Phosphate Ore Samples

phosphate ore	sample weight at [%]						
	100°C	250°C	400°C	550°C	700°C	850°C	1000°C
Algeria 1	99.853	99.313	98.139	96.713	96.078	94.261	90.362
Algeria 2	99.971	99.703	99.059	97.814	97.259	95.574	91.876
Egypt 1	99.968	99.510	98.257	97.399	96.752	95.713	94.172
Egypt 2	99.956	99.558	98.041	97.165	96.641	95.885	94.297
Egypt 3	99.953	99.451	97.811	97.187	96.862	95.633	93.510
Egypt 4	99.927	99.750	99.256	98.687	98.331	95.304	91.767
Israel 1	99.945	99.637	98.089	96.919	96.107	93.086	89.717
Israel 2	99.923	99.640	98.716	97.863	97.309	94.834	91.345
Morocco	99.969	99.679	99.145	98.235	97.657	95.452	91.822
Senegal	99.937	99.616	98.900	98.193	97.419	96.151	95.158
Togo	99.946	99.726	98.395	97.877	97.326	96.996	96.228
Tunisia	99.959	99.679	99.006	97.664	96.927	95.259	91.431

As can be seen in Figure 1, the mass loss of phosphate ore samples generally occurs in three steps. The first step is observed in the range of 40–150 °C, second in 280–410 °C, and third in 560–890 °C. These temperature ranges are highlighted in dark color in Figure 1.

Obtained results and ranges are in correlation with the findings of various previous researchers.^15,20−23^ However, most of them assigned the decomposition of organic matter as the only possible explanation for the mass loss during the second step. Although MS curves for *m*/*z* = 44 (mostly associated with the CO_2_ molecule) show some increase in this range, there are also clear peaks for *m*/*z* = 17 and *m*/*z* = 18 lines (mainly associated with the H_2_O molecule). Therefore, in our opinion, it is possible that, along with the organic matter decomposition, at least some chemical water from minerals present in the phosphate ore is released during this stage. It is very unlikely that any free water is left after the drying stage.

For the first and third steps of the mass loss of samples, the results and MS curves correspond to those obtained in the aforementioned studies. The first step mainly consists of the drying of phosphate ore, while the third is the result of the decomposition of organic matter and carbonates.

### 3.2 Calcination of the Tunisian Phosphate Ore

Phosphate ore from the Tunisian deposit was selected for further calcination studies. It has the lowest phosphate content among all tested samples (Table 1) and experiences relatively large mass loss during heating as shown by the TG-MS analysis (approximately 9%). This makes it a promising material to be processed by calcination. Additionally, this raw material has a very high economic potential. The relatively low price and high market availability make it a potential area of interest for fertilizer manufacturers.

Four samples of phosphate ore from the Tunisian deposit were prepared:

1 Tunisian phosphate ore without additives;
2 Tunisian phosphate ore with 5 wt % NaCl;
3 Tunisian phosphate ore with 5 wt % KNO_3_;
4 Tunisian phosphate ore with 5 wt % Na_2_CO_3_.

These samples were calcined at various temperatures (850, 950, and 1050 °C) for various times (1, 2, and 3 h) according to the experiment matrix of the full factorial design plan. The mass loss of each sample during calcination is presented in Table 3 and phosphate content in obtained calcinates is presented in Table 4.

**Table 3:** Mass Loss of Samples during Calcination in Various Conditions

mass loss [%]	850°C			950°C			1050°C		
	1 h	2 h	3 h	1 h	2 h	3 h	1 h	2 h	3 h
Tunisia	9.58	9.62	9.65	9.67	9.74	9.80	10.09	10.18	10.26
Tunisia + 5 wt % NaCl	8.90	9.05	9.20	9.86	10.27	10.59	12.02	12.30	12.69
Tunisia + 5 wt % KNO_3_	11.45	11.56	11.70	11.87	12.01	12.14	12.12	12.27	12.35
Tunisia + 5 wt % Na_2_CO_3_	10.87	11.10	11.31	11.26	11.36	11.49	11.49	11.56	11.62

**Table 4:** Phosphate Content in Calcinates Obtained in Various Conditions

P_2_O_5_ content in the calcinate [wt %]	850°C			950°C			1050°C		
	1 h	2 h	3 h	1 h	2 h	3 h	1 h	2 h	3 h
Tunisia	28.6	28.4	28.2	28.1	27.8	27.7	27.3	27.0	26.9
Tunisia + 5 wt % NaCl	26.8	26.8	26.6	26.7	26.7	26.5	26.4	26.2	26.0
Tunisia + 5 wt % KNO_3_	27.7	27.5	27.4	27.1	27.2	27.0	26.4	26.2	25.9
Tunisia + 5 wt % Na_2_CO_3_	27.4	27.4	27.5	27.1	26.9	26.9	26.1	25.9	25.7

Appropriate mathematical models of the mass loss of studied samples were derived using Statistica software. After rejecting the statistically nonsignificant terms for models (*p* > 0.05), the final equations are as follows
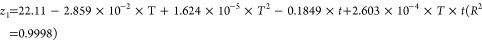
1
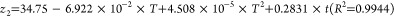
2
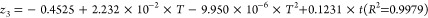
3
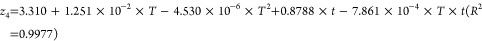
4where *z*_1_, *z*_2_, *z*_3_, and *z*_4_ are mass losses of respective samples, *T* is the temperature of calcination, and *t* is the time of calcination.

**Figure 2:**
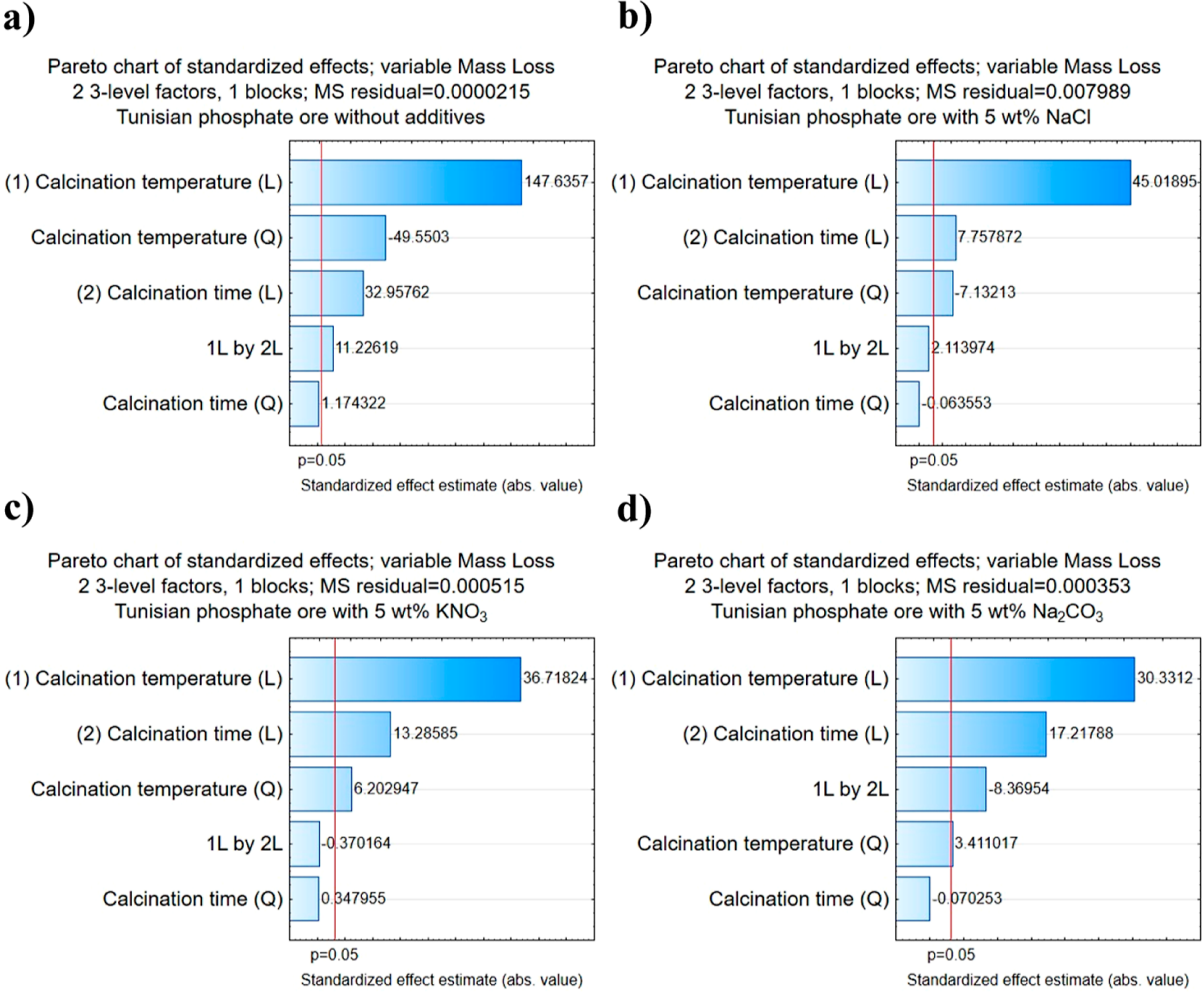
Pareto charts for conducted calcination experiments: ((a) Tunisian phosphate ore without additives; (b) Tunisian phosphate ore with 5 wt % NaCl; (c) Tunisian phosphate ore with 5 wt % KNO_3_; (d) Tunisian phosphate ore with 5 wt % Na_2_CO_3_).

Pareto charts obtained for the performed analyses are presented in Figure 2.

Obtained models for the mass loss of samples during calcination show that the calcination temperature is much more important than the calcination time. In the studied range, the mass loss of samples increases with the square of the calcination temperature and only linearly with the calcination time. In two cases (for Tunisian phosphate ore without additives and with 5 wt % Na_2_CO_3_), the interaction terms between the two variables turned out to be statistically significant as well.

**Figure 3:**
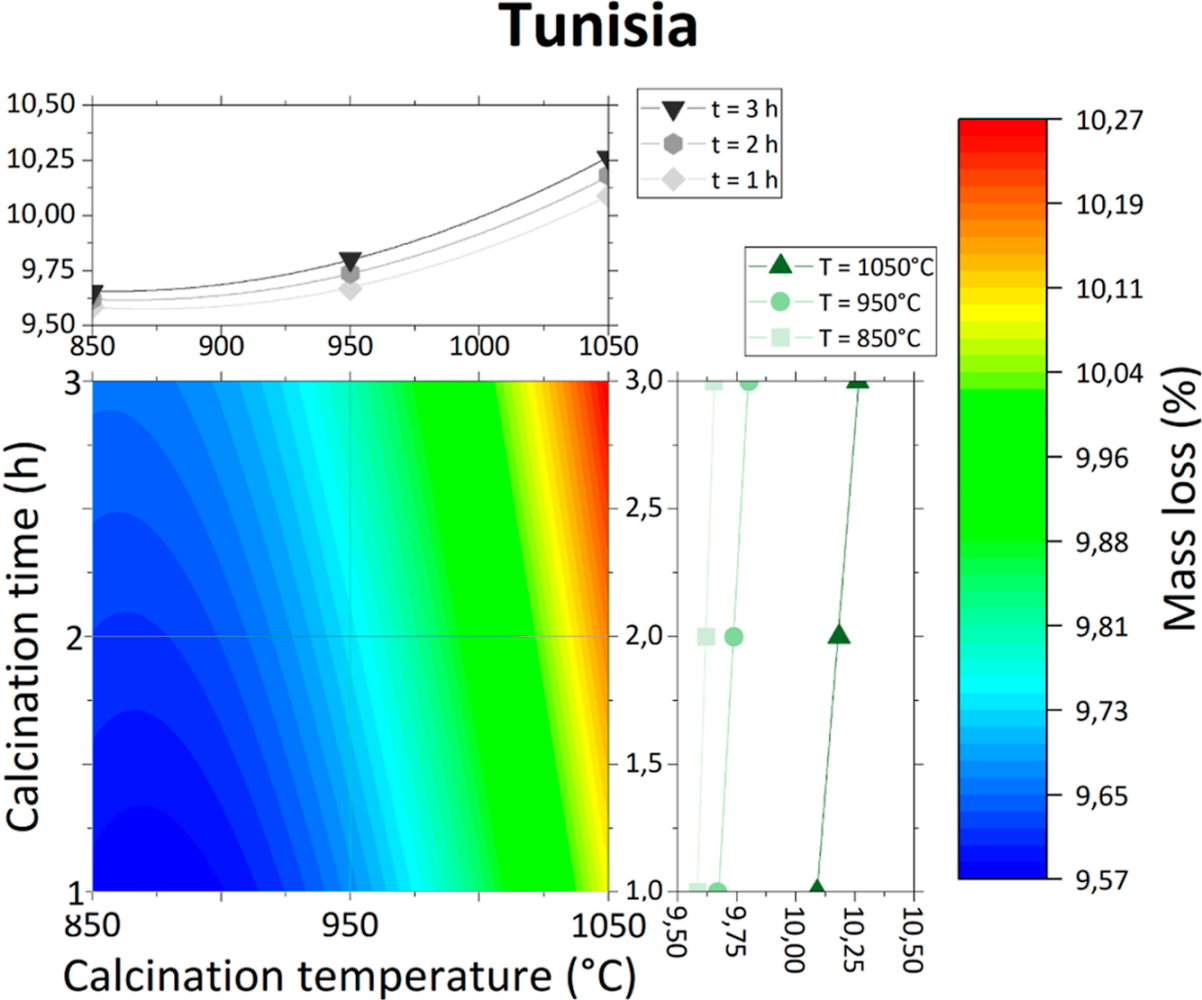
Mass loss of Tunisian phosphate ore as a function of calcination temperature and time.

**Figure 4:**
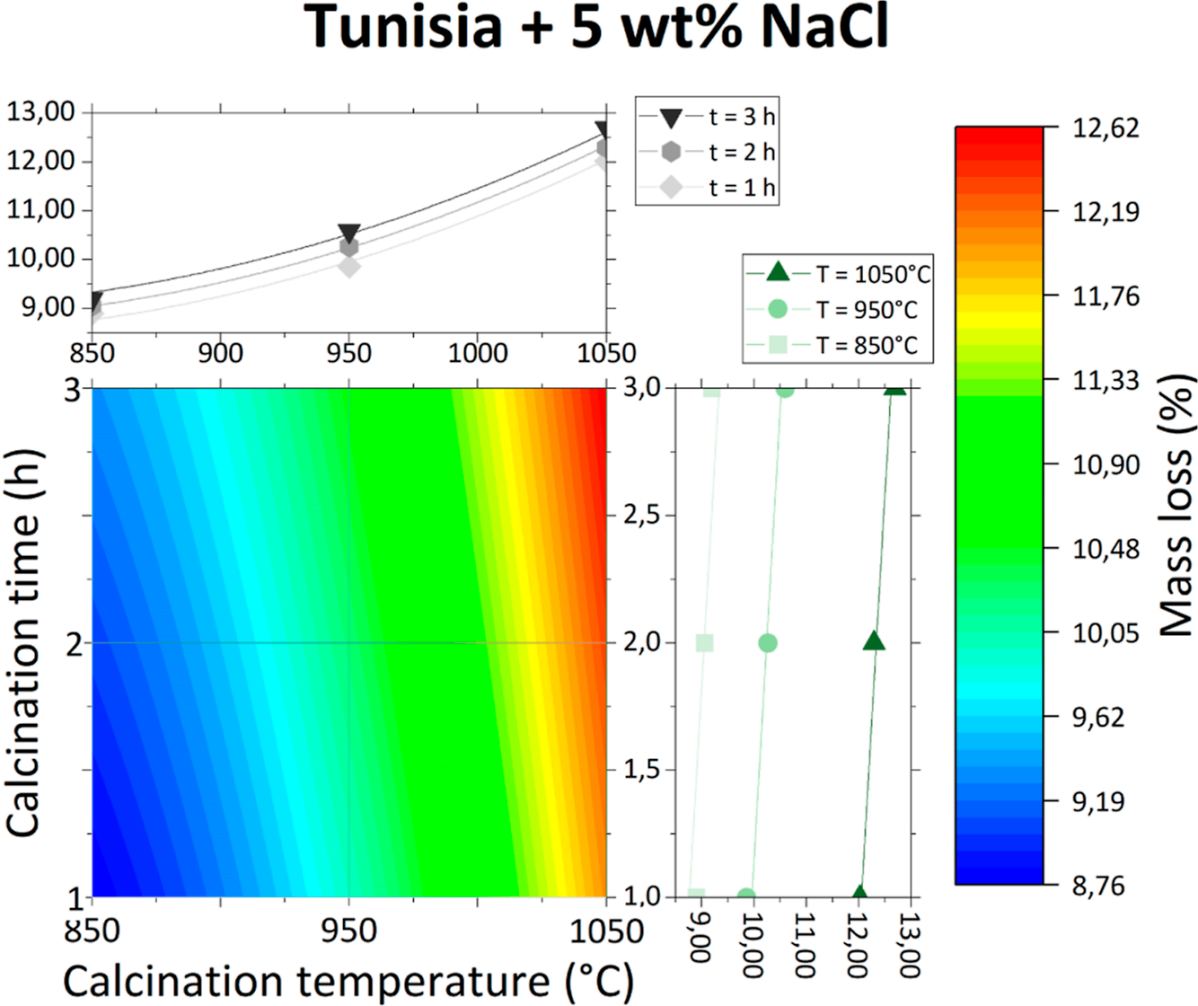
Mass loss of Tunisian phosphate ore with 5 wt % NaCl as a function of calcination temperature and time.

**Figure 5:**
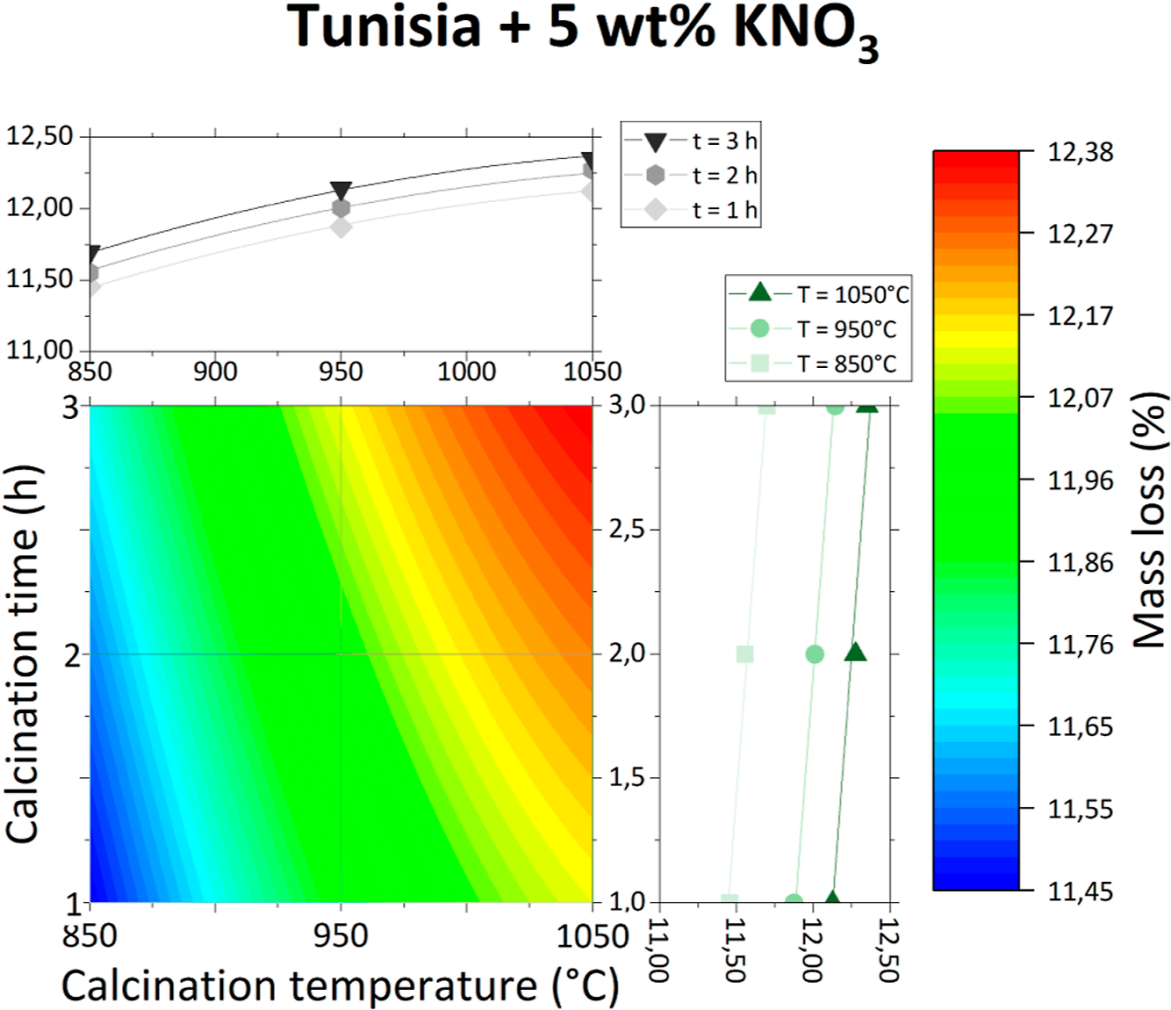
Mass loss of Tunisian phosphate ore with 5 wt % KNO_3_ as a function of calcination temperature and time.

**Figure 6:**
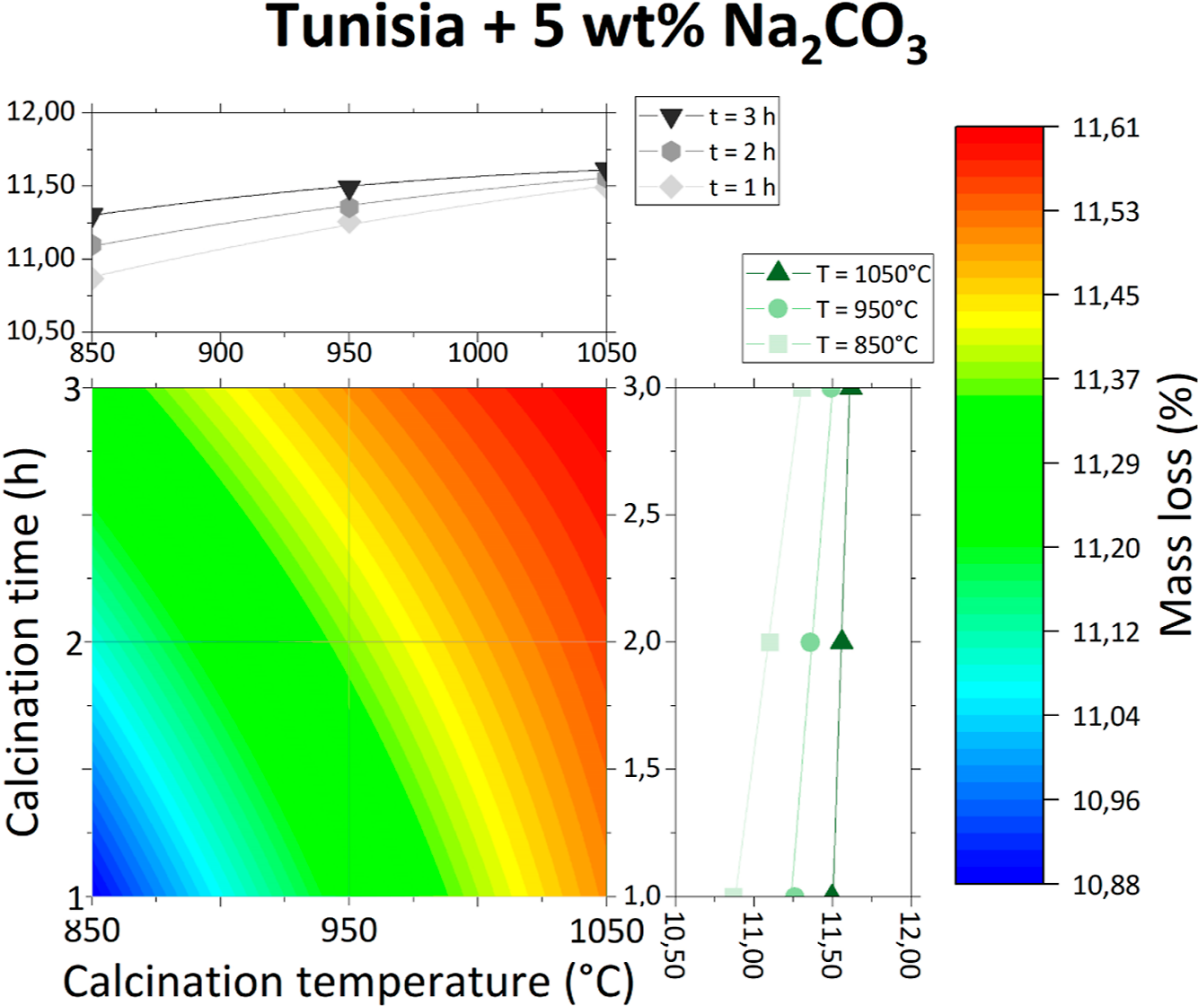
Mass loss of Tunisian phosphate ore with 5 wt % Na_2_CO_3_ as a function of calcination temperature and time.

**Figure 7:**
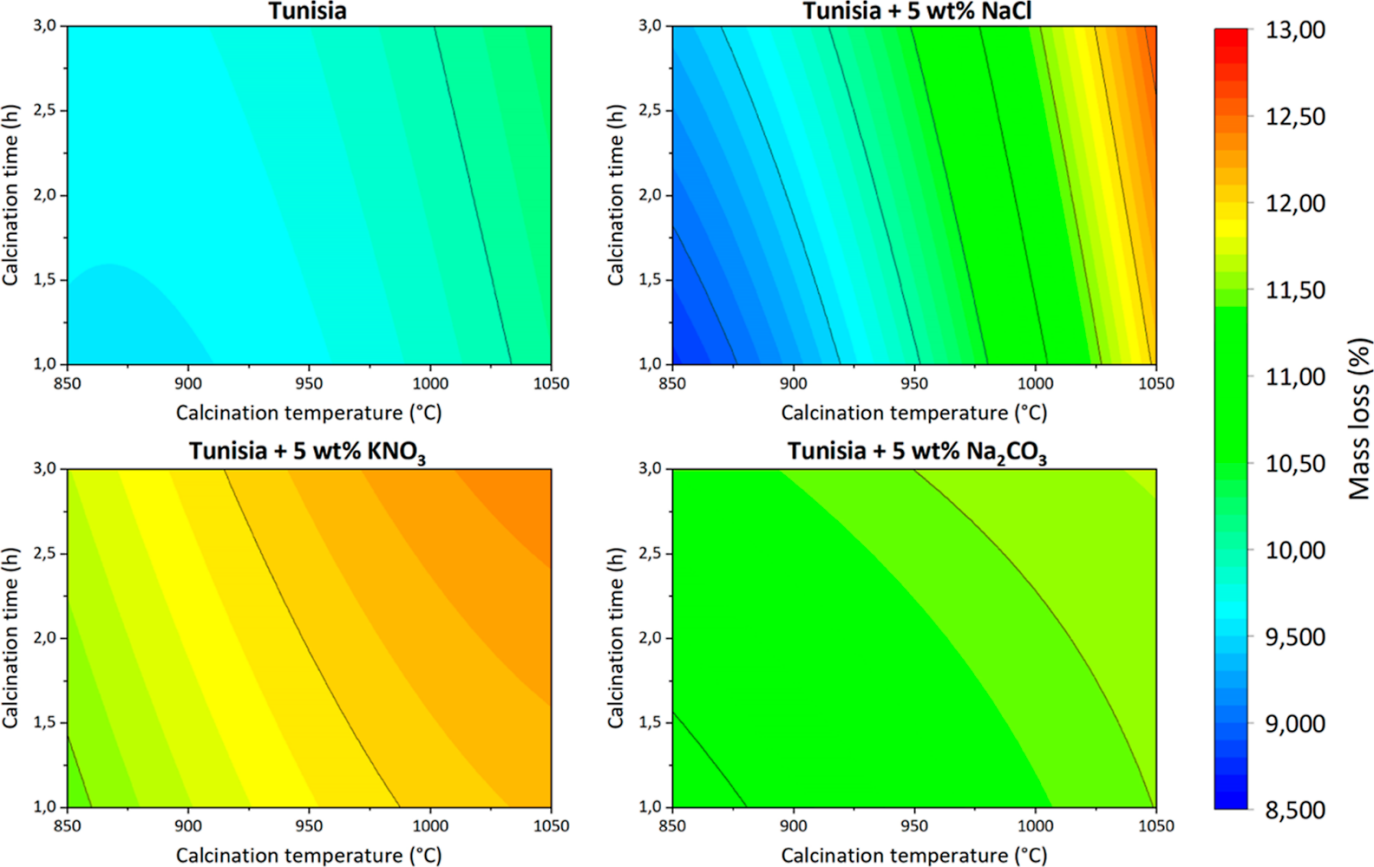
Mass loss of Tunisian phosphate ore with various additives.

Contour graphs of obtained models with profiles and experimental data points are presented individually in Figures 3–6 and together in Figure 7.

The effect of the inorganic salt addition is different for each studied salt. For both KNO_3_ and Na_2_CO_3_, the mass loss of the sample is greater than that for the phosphate ore without any additives and is only slightly variable with increasing temperature and time. This could be due to the decomposition of KNO_3_ and Na_2_CO_3_ at temperatures lower than the studied calcination temperatures. The effect of their decomposition (especially in the case of Na_2_CO_3_) on the overall mass loss is probably more important than the effect of the phosphate ore itself.

However, this is not true for the addition of NaCl, which does not decompose until very high temperatures. Therefore, at low calcination temperatures (about 850 °C), the total mass loss is lower than for the phosphate ore without any additives, as there is less water and carbonate matter in the sample coming from the phosphate ore. In higher calcination temperatures, the overall mass loss is greater than for the phosphate ore without any additives. It is possible that NaCl, after being melted (melting point approximately 801 °C) and heated, reacts with phosphate ore, producing volatile compounds.

### 3.3 Thermal Analysis of Calcined and Uncalcined Tunisian Phosphate Ore Samples

**Figure 8:**
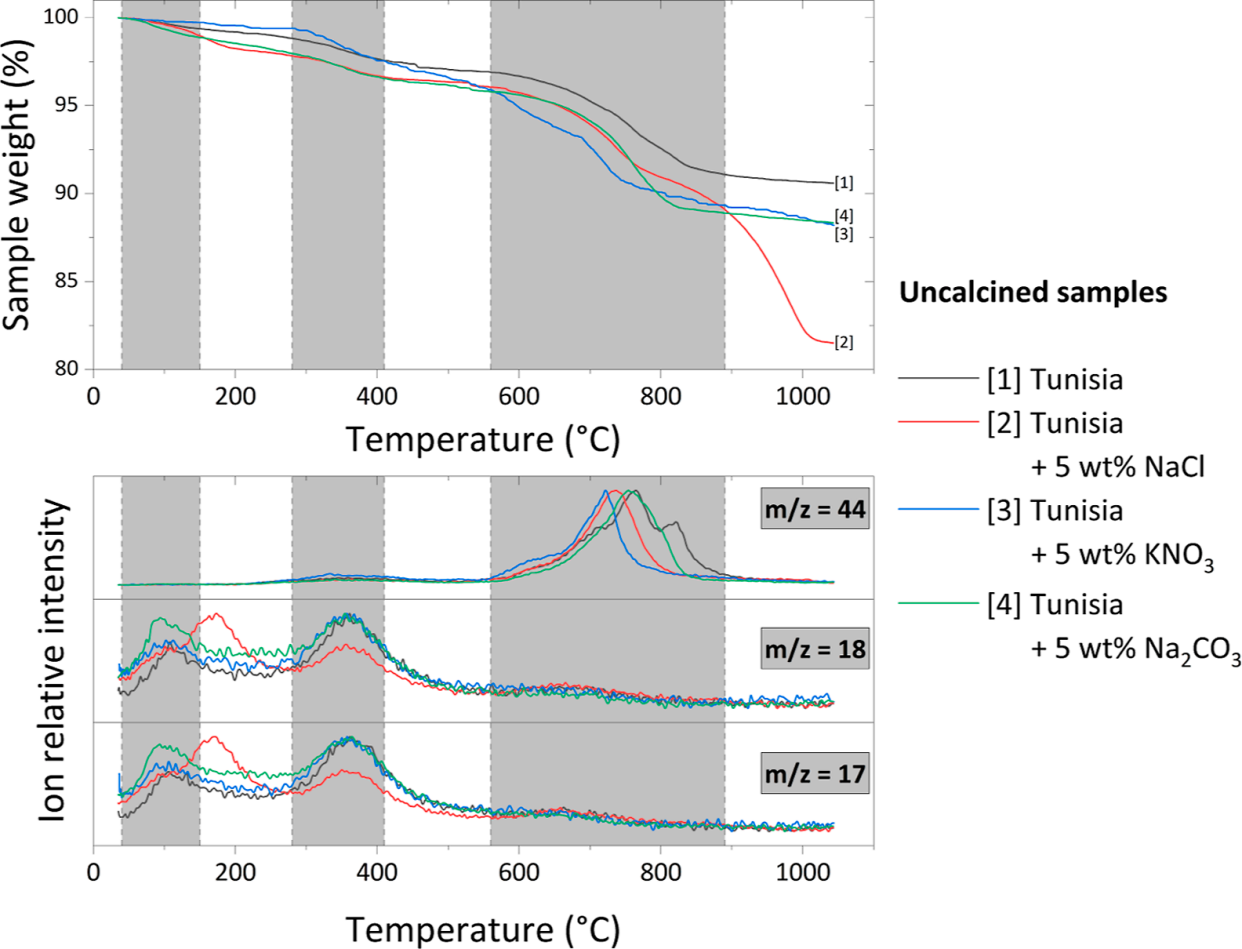
TG and MS curves for uncalcined Tunisian phosphate ore samples with various inorganic additives.

**Figure 9:**
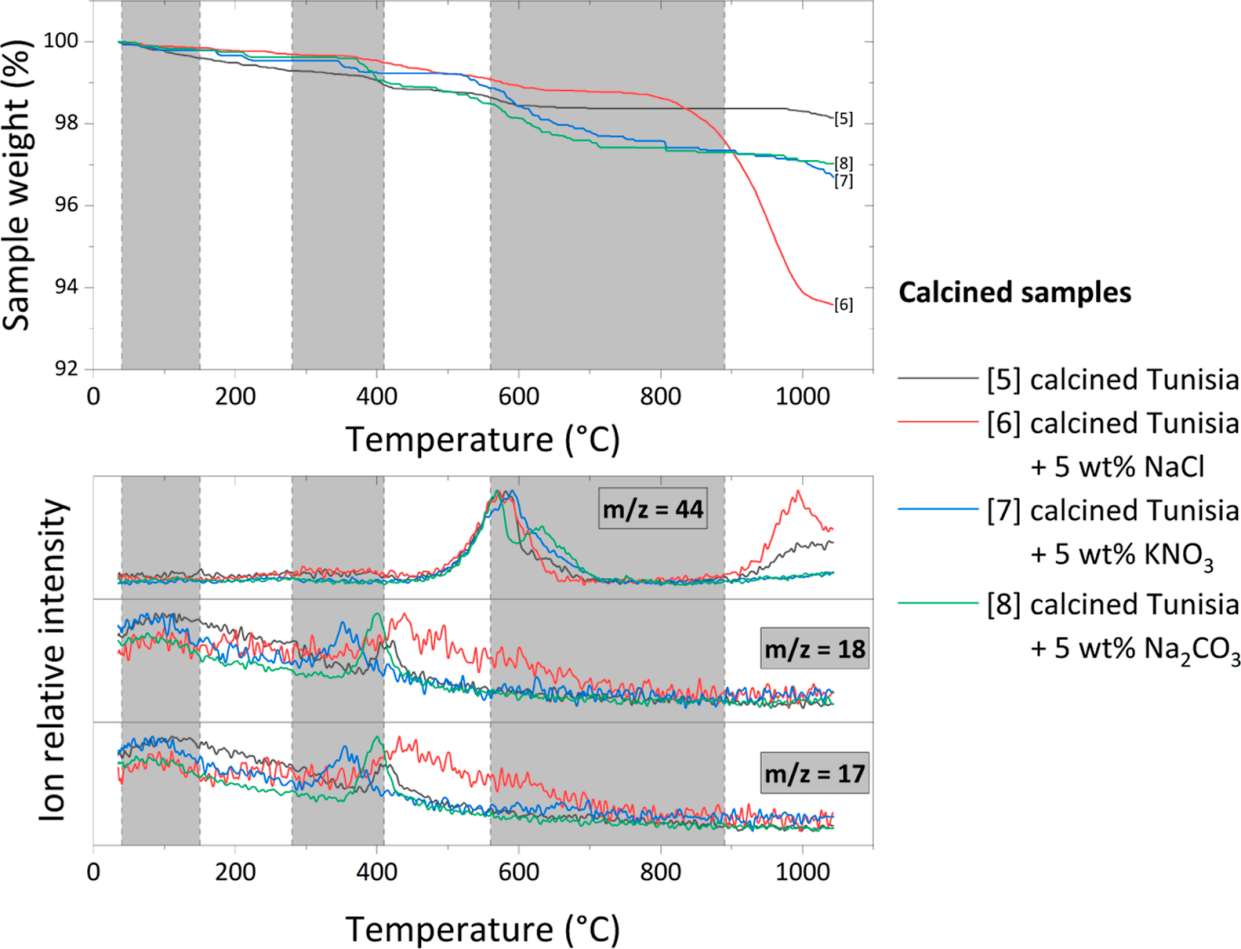
TG and MS curves for calcined Tunisian phosphate ore samples with various inorganic additives.

Samples of both calcined and uncalcined Tunisian phosphate ore with additives were subjected to TG-MS. Thermogravimetry curves, together with MS curves for *m*/*z* 17, 18, and 44, are presented in Figure 8 for uncalcined samples and in Figure 9 for calcined samples. Temperature ranges highlighted in dark color in these figures are the same as those in Figure 1.

Obtained results for uncalcined samples further support hypotheses that were formed in the previous subsection. After the addition of Na_2_CO_3_ and, especially, KNO_3_, the shape of the TG curve is very similar to that of the Tunisian phosphate ore without additives, with a slight shift. However, the shape of the curve for samples with the addition of NaCl is different. It confirms the models obtained for mass loss since there is a smaller weight loss at lower calcination temperatures (about 850 °C) than in samples containing KNO_3_ and Na_2_CO_3_. The curves cross at a temperature of about 900 °C, and from that point on, the weight loss of the sample with NaCl is much greater than for the other two additives. However, no such relation was confirmed for the sample without any additives as its weight loss was lower than for the other samples in the whole range of calcination temperatures.

Only small mass losses (about 1–2%) are observed up to 800 °C for calcined samples. Beyond that temperature, the mass of the sample with NaCl addition drops drastically, while it remains relatively stable for other samples up to 1000 °C when they start to decrease. MS curves corresponding to those measurements for *m*/*z* 17 and 18 (H_2_O molecules) show no significant peaks in the studied temperature range. This is expected because calcination occurs in high temperatures and most, if not all, of the water evaporates from the samples. Interestingly, the MS curve for *m*/*z* 44 (CO_2_ molecule) showed peaks similar to those of uncalcined samples but shifted to much lower temperatures (from about 750 °C to approximately 600 °C). Moreover, another peak is present above 950 °C, which is especially visible for the sample with NaCl addition.

In theory, calcined samples should retain their masses up to the temperature of their calcination (in this case 850 °C). However, small mass losses are observed for every studied sample. This phenomenon was also observed by Mgaidi et al.^15^ At this point, two possible explanations were proposed:

- Samples after calcination adsorb volatile compounds from the air on their surface, which are then released during another cycle of heating;
- Samples were not fully calcined during the process.

To verify the first hypothesis, one of the samples after the TG-MS measurement was left in the crucible for 48 h under ambient conditions. After that, the measurement was repeated, and this time, no mass loss was detected. This hypothesis is further rejected by the results of previous investigators, who determined that the surface area of phosphate ore is lower after the calcination process.^12,15^ It is very unlikely that the calcinate would adsorb any substance, let alone any substance, from the air.

Therefore, it was assumed that the second hypothesis is correct. During calcination, the amount of sample was greater than the amount needed to cover the bottom of the crucible during thermal analysis. An excessive amount of sample formed layers on top of the heated mass. After this process, hard but very brittle sinters were formed. It is possible that this did not allow all volatile compounds to escape, and some were trapped inside the resulting sinter. This was not the case for the TG-MS measurement. This method requires very little sample for analysis, and therefore, it can be assumed that it was monolayered in the alumina crucible, allowing for complete calcination.

## 4 Conclusions

Phosphate ores lose weight in three steps during heating to elevated temperatures. The first step is in the range of 40–150 °C and corresponds to drying of the sample and loss of free water. The second step is in the range of 280–410 °C and indicates the decomposition of organic matter and/or loss of some chemical water from minerals present in the phosphate ore. The third step is in the range of 560–890 °C and relates to organic matter and carbonate decomposition.

Therefore, the calcination of phosphate ore can be a viable method to process the ore, i.e., to remove some unnecessary substances that are only a part of the phosphate ore matrix. This process can be further improved by the addition of inorganic salts mixed with the phosphate ore, as their presence could lower the required calcination temperature and, in turn, allow the significantly lower high costs of such processes. All inorganic salts used in this study as additives have good solubility in water and could be easily removed from the product by washing the crushed calcinate with water. In an example experiment, the sample of phosphate ore with 5 wt % NaCl calcined for 2 h at 950 °C (middle sample) was washed with water in a 1:1 (m/m) ratio. The sample after calcination contained 0.94 wt % Na_2_O before washing and 0.11 wt % Na_2_O after washing.

From the studied salts, NaCl seems to have the greatest potential as a beneficial additive to the phosphate ore. It allows a great increase in the weight loss of the ore, compared to the sample without any additive, at the same or even lower calcination temperature.
